# Endothelin receptor antagonism for resistant hypertension in chronic kidney disease: where does aprocitentan fit?

**DOI:** 10.1093/ckj/sfag289

**Published:** 2026-08-26

**Authors:** Emelina Stambolliu, Carmine Zoccali

**Affiliations:** Nephrology Department, Hippokration General Hospital, Athens, Greece; Renal Research Institute, New York, NY, USA; Institute of Molecular Biology and Genetics, Ariano Irpino, Italy; Associazione Ipertensione Nefrologia Trapianto Renale, Reggio Calabria, Italy

Resistant hypertension, defined as blood pressure (BP) above target despite maximally tolerated doses of at least three antihypertensive agents, including a diuretic, after excluding pseudo-resistance, is common in chronic kidney disease (CKD), and is associated with persistently low rates of BP control [1, 2]. Uncontrolled hypertension contributes to the elevated cardiovascular morbidity and mortality seen in this population, hence merits aggressive and sustained management [2].

Hypertension in CKD is multifactorial, reflecting volume expansion, neurohormonal activation, vascular stiffness, and endothelial dysfunction, hence therapeutic strategies beyond conventional renin–angiotensin system (RAS) blockade and diuretics remain attractive. Among these, the endothelin (ET) pathway is particularly relevant, given its role in vasoconstriction, sodium handling, inflammation, fibrosis, and proteinuria. Indeed, ET-pathway modulators have already shown efficacy in glomerulonephritis [3].

Aprocitentan, a dual ETA/ETB receptor antagonist, demonstrated efficacy for resistant hypertension in the phase 3 PRECISION trial [4]. A post hoc analysis of this 48-week trial assessed its efficacy and safety specifically in participants with kidney impairment [5].

The trial included participants with office systolic BP ≥140 mmHg (trough, unattended), eGFR ≥15 ml/min/1.73 m², and NT-proBNP <500 pg/ml. Participants received fixed-dose triple-combination background therapy (amlodipine 5 or 10 mg/valsartan 160 mg/hydrochlorothiazide 25 mg) before randomization to aprocitentan 12.5 mg, 25 mg, or placebo [4, 5].

This post hoc analysis included 147 participants (20.1% of the total sample) with kidney impairment, defined as baseline eGFR <60 ml/min/1.73 m², urinary albumin/creatinine ratio (UACR) ≥30 mg/g, or both. Participants’ baseline characteristics were: 70.7% male, mean age 66.1 years, 91.7% with UACR >30 mg/g, 76.2% with CKD stage 3–4, and 71.4% on ≥4 antihypertensive medications [5]. Results of the analysis are summarized in Table 1.

**Table 1:** Summary of the post hoc analysis findings (*N* = 147).

Efficacy outcomes			
Variable	Aprocitentan 12.5 mg	Aprocitentan 25 mg	Placebo
PART 1: Baseline to week 4			
Office SBP, mmHg	−13.5 (2.3)	−16.6 (1.9)	−4.4 (2.9)
Daytime ABPM SBP, mmHg	−8.5 (2.2)	−7.9 (2.6)	+2.7 (3.1)
Nighttime ABPM SBP, mmHg	−9.6 (2.1)	−13.8 (2.8)	−2.5 (3.1)
UACR, %	−47.1% (−56.1 to −36.4)	−59.6% (−69.1 to −47.1)	−2.4% (−24.4 to 26.1)
eGFR, ml/min/1.73m²	+0.5 (8.1)	−2.5 (8.4)	−0.4 (8.2)
Hemoglobin, g/l	−10.2 (8.9)	−10.3 (6.8)	−2.0 (7.1)
PART 2: Week 4 to week 36 (all on Aprocitentan 25 mg)			
Office SBP, mmHg	−16.4 (1.9)		
UACR, %	−61.6% (−70.2 to −50.7)		
Hemoglobin, g/l	−11.8 (8.6)		
eGFR, ml/min/1.73m²/year	−0.9 (−4.0 to 2.2)		
PART 3: Week 36 to week 40 (re-randomized to Aprocitentan 25 mg or placebo)			
Office SBP, mmHg	−0.8 (2.0)		+5.5 (2.7)
Daytime ABPM SBP, mmHg	−2.3 (2.5)		+1.4 (1.9)
Nighttime ABPM SBP, mmHg	0.0 (2.0)		+10.3 (2.2)
UACR, %	−12.9% (−26.1 to 2.7)		+108.3% (46.0–197.1)
eGFR, ml/min/1.73m²	−1.0 (9.4)		−1.1 (9.1)
Hemoglobin, g/l	0.0 (6.3)		+9.2 (10.0)
Safety outcomes			
Event	Finding		
Edema	37.9% (55/145); mostly mild/moderate peripheral leg edema, effectively managed with/without added diuretic, discontinuation due to edema 2.1% (3/145)		
Heart failure events	11 participants (7 hospitalized); none treatment-related, none led to drug discontinuation		

Results are presented as within-group mean change from baseline (for Part 1 and Part 2, while for Part 3 the “baseline” is Week 36), with SE or 95% CI, unless otherwise indicated.

SE, standard error; CI, confidence intervals; SBP, systolic blood pressure; ABPM, ambulatory blood pressure monitoring; and UACR, urinary albumin/creatinine ratio.

This post hoc analysis provides the first focused assessment of aprocitentan’s efficacy and safety in patients with kidney impairment, defined by reduced eGFR, albuminuria, or both. Its BP-lowering effect on both daytime and nighttime ambulatory BP, up to 8.5 and 13.5 mmHg, respectively, was notable, and UACR reduction was also substantial, approaching 60% in the short-term randomized phase with further reduction during continued exposure, while eGFR remained broadly stable over follow-up; however, these findings should be considered hypothesis-generating rather than evidence of renal protection. Concerning safety, the principal adverse event was edema, which was generally manageable and rarely led to drug discontinuation [5]. This signal is biologically plausible, as ET receptor antagonism can promote sodium and water retention, making volume assessment and diuretic optimization central to safe use.

Why should aprocitentan be considered as a fourth- or fifth-line antihypertensive agent when other options already exist? At present, no data demonstrate that aprocitentan improves hard cardiovascular or kidney outcomes beyond its BP-lowering effect. Spironolactone remains the benchmark fourth-line therapy for resistant hypertension, based largely on PATHWAY-2, but hyperkalemia substantially limits its use in advanced CKD, particularly in patients receiving RAS blockade [6]. In this context, aprocitentan may offer a mechanistically distinct alternative. Its effect on nighttime BP is particularly noteworthy given the prognostic importance of nocturnal hypertension [7], although whether this translates into long-term clinical benefit remains unknown.

Achieving and maintaining euvolemia is likely to be a prerequisite for safe aprocitentan use, and concomitant diuretic therapy may be necessary. This could complicate treatment in advanced CKD, where volume status is often unstable. Anemia also deserves attention. The observed hemoglobin reductions may reflect hemodilution related to fluid retention, but in CKD even modest decreases could aggravate pre-existing renal anemia or complicate erythropoiesis-stimulating agent management. Finally, this remains a post hoc, non-prespecified analysis, not prospectively powered to assess CKD-specific efficacy or safety, and findings should be interpreted accordingly.

Taken together, aprocitentan appears to be a promising addition to the therapeutic armamentarium for resistant hypertension in CKD, particularly for patients in whom spironolactone is poorly tolerated or contraindicated. Its place in practice, however, will depend on careful patient selection, volume management, monitoring for edema and anemia, and ultimately on whether BP and albuminuria reductions translate into improved cardiovascular or kidney outcomes.
